# The Influence of Processing and Particle Size on Binderless Particleboards Made from *Arundo donax* L. Rhizome

**DOI:** 10.3390/polym12030696

**Published:** 2020-03-21

**Authors:** Manuel Ferrandez-Villena, Clara Eugenia Ferrandez-Garcia, Teresa Garcia-Ortuño, Antonio Ferrandez-Garcia, Maria Teresa Ferrandez-Garcia

**Affiliations:** Department of Engineering, Universidad Miguel Hernandez, 03300 Orihuela, Spain; cferrandez@umh.es (C.E.F.-G.); tgarcia@umh.es (T.G.-O.); antonio.ferrandezg@umh.es (A.F.-G.); mt.ferrandez@umh.es (M.T.F.-G.)

**Keywords:** waste material, panels, physical and mechanical properties, giant red

## Abstract

The giant reed (*Arundo donax* L.) is considered one of the world’s 100 worst invasive species. The main method by which this species propagates is by growth of scattered fragments of rhizome, spreading without control with very strong, deep roots. Agricultural waste consists of lignocellulosic materials that can substitute natural wood and offer a suitable alternative with which to manufacture boards for furniture, packaging and building purposes. The objectives of this work were to obtain binderless particleboards using giant reed rhizome as the raw material, to evaluate their mechanical and physical properties according to the applicable European standards and to assess the self-binding mechanism of the particles in the board. Six types of boards (12 classes) were manufactured with giant reed rhizome biomass. They were manufactured with a temperature of 110 °C, a pressure of 2.5 MPa and pressing times of 7 and 15 min, applying one or two pressing cycles. The results achieved for modulus of rupture (14.2 N/mm^2^), modulus of elasticity (2052.45 N/mm^2^) and internal bonding strength (1.12 N/mm^2^) show that the mechanical properties were improved by using a smaller rhizome particle size and two pressing cycles.

## 1. Introduction

The giant reed (*Arundo donax* L.) is considered one of the world’s 100 most invasive species, according to the UICN’s Global Invasive Species Database, and it is also included in the Spanish Catalogue of Invasive Species [1]. Its root system is a thick, knotted rhizome, with very strong, deep (150 cm), adventitious roots. The main method by which this species spreads is by growth of scattered fragments of rhizome. In river beds, the force of the water sections the rhizomes and acts as the dispersing agent. Due to the rapid rate of growth and vegetative reproduction of this species, it occupies new areas and forms dense masses (reed beds), causing a profound transformation of the ecosystems that it invades at the expense of other native species.

In order to solve the problems caused by this invasive species and attempt to recover the ecosystem, the methods that have been used to control it are the application of glyphosate [2], the use of mechanical cutting and extraction tasks and the use of biological control measures [3], but none of these techniques have been effective [4]. The rhizomes are capable of germinating regardless of their size and in most environmental conditions, since they are highly tolerant to saline conditions and can adapt well to the accumulation of salt in the soil [5]. Therefore, when the rhizomes are pulled up and shredded, in the areas where this material is deposited the reeds grow back and spread without control to other places [6].

Due to its high biomass yield, its adaptation to different soil types and weather conditions, its lower tillage requirements than traditional crops, its phytoremediation properties [7,8,9] and its efficiency as a biofilter for wastewater treatment [10], the giant reed has been considered a competitive energy crop [11]. It has been proposed that this part of the giant reed could be used for manufacturing paper [12], extracting xylose [13], producing activated carbon [14], processing compost [15], generating biomass [16,17], producing biogas [18], obtaining biofuel [19,20,21], forming lignocellulosic films [22], preparing composites [23], designing reinforcements for cement mortar [24] and constructing particleboards [25].

In analyses carried out of the rhizome’s components [26] non-structural carbohydrates (NSCs) were the highest component weight fraction of the dry matter (DM). Sucrose was the main NSC in the rhizome, representing on average 87% of all soluble carbohydrates and 67% of total NSCs. Other soluble sugars found in measurable quantities in all samples were glucose, fructose and raffinose, but these were found in low amounts (close to 1% of the DM as a total). The starch content was considerable and it varied according to the area from it was taken, representing between 12.7% and 39.8% of NSCs [26].

Concern for the environment and for energy efficiency has also led to many different proposals in the field of construction in order to meet users’ demands. Currently, the use of natural resources is rationalised and the application of building materials manufactured with plant fibres is increasing, as these products are not considered harmful to the environment.

There is also a great deal of interest in manufacturing formaldehyde-free boards, meaning that there is increasing pressure on particleboard producers to stop using these binders. In this regard, studies have been carried out on particleboards in which synthetic resins are replaced with natural resins and adhesives, such as protein, lignin, tannin, gluten, starch, citric acid, etc. [27,28,29,30,31,32,33].

At present, research with plant biomass is aimed at producing binderless particleboards with different pre-treatments; the self-binding capacity of natural fibres on reaching the glass transition temperature has been amply demonstrated. The size and shape of the particle can significantly affect the properties of binderless boards [34]; therefore, determining the range of particle sizes is an important parameter for improving their binding capacity. The starting material for manufacturing binderless particleboards contains cellulose, hemicellulose, lignin and also polysaccharides; we therefore have good expectations of the self-binding capacity of its natural fibres as these compounds are degraded on reaching the glass transition temperature, although it is not known exactly how each component contributes to the self-binding of the particles [35].

By increasing the temperature to above 180 °C, using longer pressing times and applying greater pressure than that used in conventional industry, the mechanical strength and stability of binderless particleboards have been improved [36]. These parameters have the great disadvantage of significantly increasing the energy consumption of the process, so these are not considered recommendable working parameters.

Tests carried out with binderless giant reed boards showed that very poor physical and mechanical properties were obtained [32], but as the giant reed rhizome has a large amount of fermentable sugars [26], it can be surmised that this material could be used to produce binderless boards with good properties.

Bearing in mind the need for new materials made from reused waste, the objective of this work is to manufacture and evaluate binderless particleboards made from giant reed rhizome, analysing the self-binding mechanism of the particles in the board.

## 2. Materials and Methods

### 2.1. Materials

The materials used were giant reed rhizomes and water from the municipal mains water supply. The giant reed rhizomes came from clearing the bed of the River Segura, in Southeast Spain. The rhizomes were left to air dry for 5 months before being shredded in a blade mill. The particles obtained were then separated in a vibrating sieve and classified according to the sieve that they passed through and the sieve in which they were retained. The humidity of the particles was approximately 15%, and they were left to air dry until they reached a humidity of 9%. The boards were manufactured using three particle sizes (0.25 to 1 mm, 1 to 2 mm and 2 to 4 mm).

### 2.2. Methods

#### 2.2.1. Particleboard Manufacture

The boards were manufactured in a 400 mm × 600 mm mould at a temperature of 110 °C and a pressure of 2.5 MPa. These parameters were selected in order to obtain a product that has advantages in terms of energy consumption [37] with respect to those used in industrial board production, which usually involves applying temperatures between 180 and 200 °C and pressures of almost 3.5 MPa. High temperature and pressure values significantly increase the energy consumed during the process, so this line of action is not considered recommendable [34].

The manufacturing process with one cycle consisted of putting the particle mixture into the mould and then spraying the surface with water (10% of the weight of the particles) and placing it in the hot plate press. The surface of the boards that were subjected to a second pressing cycle was sprayed with water (5% by weight) before being subjected to pressure and heat.

**Figure 1:**
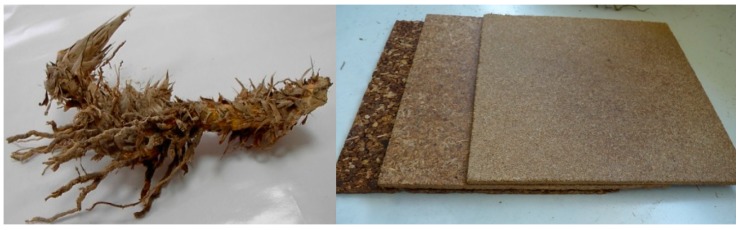
*Arundo donax* L. rhizome and binderless rhizome particleboards.

Twelve classes of boards were manufactured, with an approximate thickness of 10 mm, using three particle sizes and two pressing times, with either one pressing cycle (7 and 15 min) or two pressing cycles (7 + 7 min and 15 + 15 min). Table 1 shows the manufacturing characteristics of each class of board manufactured, and Figure 1 shows some manufactured rhizome particleboards.

The mean thickness of the boards was 7 mm, and they were subsequently cut into samples of the appropriate dimensions, as specified in the European standards for each of the laboratory tests. The samples were kept for 24 h in a conservation chamber, at a temperature of 20 °C and a relative humidity of 65%, before the tests were performed.

#### 2.2.2. Experimental Tests

The method followed was experimental, with tests conducted in the Materials Strength Laboratory. The values were determined according to the European standards established for wood particleboards. Before performing the tests, samples of each board were cut and placed in a conservation chamber at a temperature of 20 °C and 65% relative humidity.

The density [38], thickness swelling (TS) and water absorption (WA) after 2 and 24 h immersed in water [39], modulus of rupture (MOR) and modulus of elasticity (MOE) [40], and internal bonding strength (IB) [41] were measured. The European standards were applied in order to evaluate the boards [42].

The mechanical tests were performed with the IMAL testing machine (Model IB700, IMAL, S.R.L., Modena, Italy), which complies with the required velocity for each test, as specified in the applicable European standards.

Morphology of the interior of the three raw materials was evaluated by using a scanning electron microscope (SEM), and an elemental analysis (quantitative and semi-quantitative) was conducted by using energy-dispersive X-ray spectroscopy (EDS). Pictures were taken from fractured cross sections of 5 × 5 mm. For the observation, a microscope (Hitachi S3000N) equipped with an X-ray detector (Briuke XFlash 3001) (Hitachi, Ltd., Tokyo, Japan) was used.

The standard deviation was obtained for the mean values of the tests and analysis of variance (ANOVA) was performed. The statistical analyses were performed using SPSS v.26.0 (IBM, Chicago, IL, USA) software from IBM.

## 3. Results and Discussion

### 3.1. Physical Properties

The physical properties obtained are shown in Table 2.

#### 3.1.1. Density

The density of the boards ranged between 735.25 and 912.76 kg/m^3^, so they can be considered medium-density boards [43]. Having performed the analysis of variance (Table 3), it can be observed that the density depends on the particle size and the pressing cycle but does not depend on the pressing time, obtaining higher values with a smaller particle size and a second pressing cycle; particle size is the manufacturing variable that has the most impact. This result is in line with that obtained in other studies with binderless boards [32], which state that density depends on the particle shape and size; to reduce the density, it is necessary to take particle size and shape into account.

#### 3.1.2. Thickness Swelling

**Figure 2:**
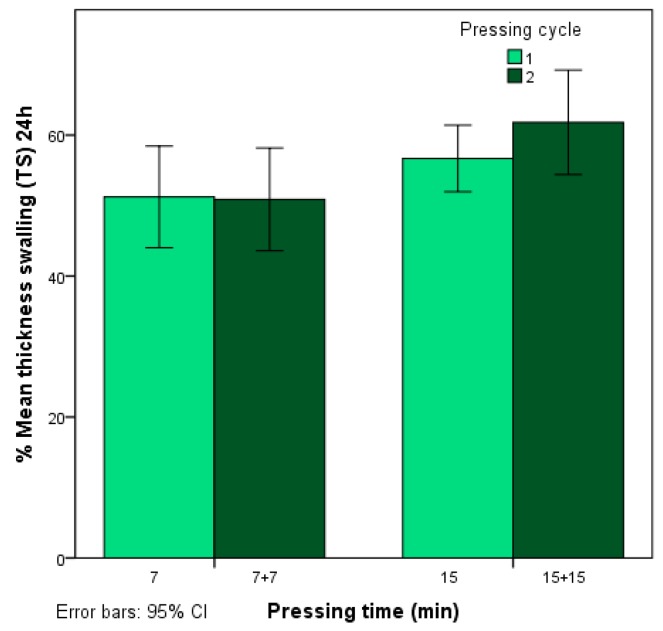
% Thickness swelling after 24 h (TS) according to pressing time.

The mean thickness swelling (TS) values in % after 2 h and 24 h immersed in water are shown in Table 2, where it can be observed that very high measurements, between 44.15% and 72.50%, are achieved after 24 h. According to the ANOVA (Table 3), the TS depends on the time in the hot plate press but is not significantly influenced by the particle size used or the pressing cycle. Figure 2 shows the TS values obtained after 24 h according to the time in the hot plate press, and it can be seen that a longer pressing time results in greater TS values.

#### 3.1.3. Water Absorption

The mean water absorption (WA) values in % are shown in Table 2. The rhizome boards absorb a large amount of water and their parameters after 24 h range between 67.09% and 106.12%. The statistical analysis (Table 3) indicates that the WA depends on the time in the press; that is, a longer pressing time results in an increase in the WA values. However, the particle size used and the pressing cycle applied have no influence in this case.

Regardless of the particle size used, the TS and WA values are very high, which can be explained by the high porosity that exists in all the types of board. Pressing time strongly influences TS and WA, as shown by other research on binderless boards [34,36], so stability can be improved by increasing the pressing time. These parameters can also be improved by increasing the application temperature during manufacture of the boards, as stated in other studies [35].

### 3.2. Mechanical Properties

#### 3.2.1. Modulus of Rupture

**Figure 3:**
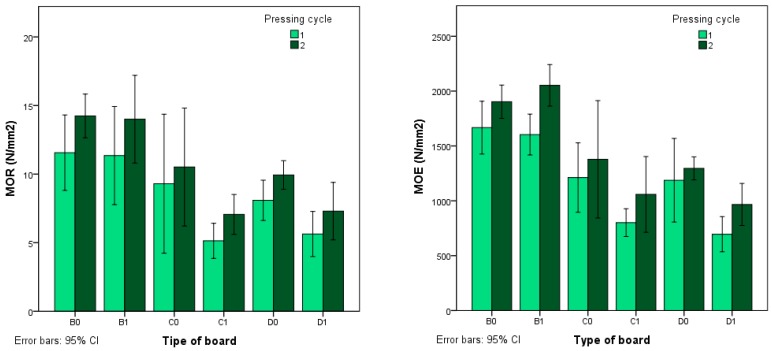
Modulus of rupture (MOR) and modulus of elasticity (MOE) according to type of board.

As can be seen in Figure 3, a greater modulus of rupture (MOR) is achieved when the boards are subjected to a second pressing cycle and with a smaller particle size. Pressing time also affects the MOR, achieving greater MOR values in the boards with a shorter pressing time.

Likewise, the same behaviour is observed in the modulus of elasticity (MOE) values, as can be seen in Figure 3. The ANOVA (Table 3) shows that the MOR and MOE depend on the particle size, pressing time and pressing cycle.

#### 3.2.2. Internal Bonding Strength

**Figure 4:**
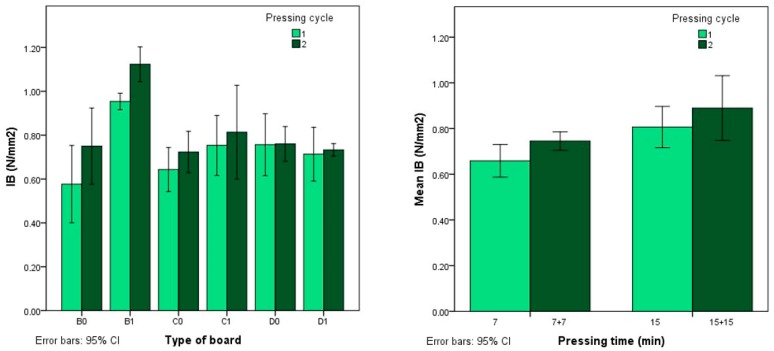
Internal bonding strength (IB) according to type of board and pressing time.

As shown in Figure 4, very high values are obtained, ranging from 0.58 to 1.12 N/mm^2^. The analysis shows that these depend on the pressing time and cycles used. In Figure 4 it can be seen that the IB increases with a longer pressing time; this is also the case when the boards are subjected to a second pressing cycle.

### 3.3. SEM Observations and EDS Analysis

In *Arundo donax* L. rhizome, the epidermis is formed by thin-walled quadrangular cells. Cortex presents layers of thin-walled cells of parenchyma, where there are randomly distributed concentric amphivasal vascular bundles.

**Figure 5:**
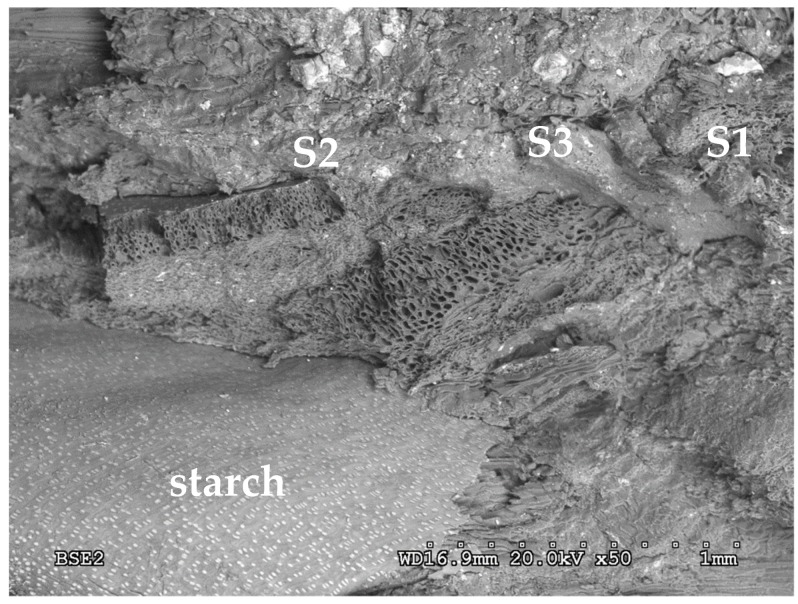
Micrograph of rhizome cross-section.

There are structures in the parenchyma that contain different chemical elements retained in the rhizome (silicon, potassium, phosphorus, magnesium, etc.), as shown in Table 4 for the sections indicated in Figure 5. This composition confirms why the cultivation of giant reed has been proposed for the functional improvement and recovery of contaminated soils (phytoremediation) [7].

The innermost layer of cortical parenchyma has abundant starch granules, as shown in Figure 5.

It can be observed in the chemical composition of different sections of the rhizome (Table 4) that the highest fractions in terms of dry matter weight are carbon and oxygen, showing the large proportion of carbohydrates contained in the rhizome. This result is consistent with the results obtained in other studies [26].

Silicon is one of the elements that is present in a larger amount and it may help bind the particles in the board [44], but this result is not conclusive. The rhizome also contains Ca and C, which can influence how the boards behave in the presence of water [45], as the addition of CaCl_2_ to wheat straw particles resulted in a 25% decrease in the WA of the particleboards manufactured.

**Figure 6:**
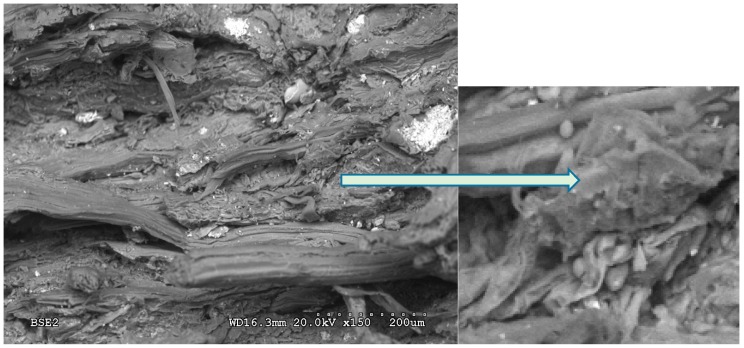
Fragment of class B01 rhizome board. Starch granules.

The micrograph in Figure 6 shows the bonds between the particles in the class B01 particleboard, particularly the existence of small non-gelatinised starch granules. The reasons why the starch is not completely gelatinised are a lack of water in the production of the board, the low temperature applied (110 °C) and the fact that 7 min in the hot plate press was not long enough. Better mechanical properties were achieved in the boards manufactured with a longer pressing time or a second pressing cycle, which shows that there has been significantly more gelatinisation of the starch.

### 3.4. Discussion

Of the three board manufacturing variables, the particle size affects density, MOR, MOE and IB; the pressing time affects MOR, IB, TS and WA; and the pressing cycle affects MOR, MOE and IB. The particle size for manufacturing the boards has become the most important factor in the tests performed by the investigators, and it can be concluded that better properties are achieved with a smaller particle size [46], as shown in this work.

Other authors [36] state that better properties are achieved by increasing the pressing time, but in this work, less swelling and good MOR and MOE values are achieved with two pressing cycles and a shorter time in the hot plate press.

Most of the studies consulted [35] state that high temperatures, above 180 °C, are needed to manufacture binderless particleboards. However, the giant reed rhizome particleboards produced in this work were manufactured at a temperature of 110 °C and could be used as stipulated in the European standard specifications [42].

If we compare the values obtained for giant reed rhizome particleboards and the values specified in the standard [42], which determine the possible compatible uses of the boards (Table 5), some would be categorised as type P1 (general purpose boards for use in dry conditions) and others would be classified as type P2 (boards for interior fitments including furniture). None of them could be designated as type P3 (non-structural boards for use in humid conditions), since they do not meet the four requirements, but the application of water-repellent substances could be considered in future works in order to reduce the TS.

In order to evaluate the boards manufactured, Table 6 gives a comparison of the values achieved in this work and the results obtained by other authors, showing that with a low temperature and short pressing time it has been possible to manufacture giant reed rhizome particleboards with better properties than with the other materials that have been investigated.

Giant reed rhizomes have a high concentration of water-soluble fermentable sugars [26]. The process by which self-binding of the particles occurs may be because the addition of water at pressing temperatures of 110 °C transformed the sugars into furfural, thus favouring the bonding mechanism of the particles. The high starch content also contributes to particle binding [32], although the temperature should be increased or water should be added to ensure proper gelatinisation. When water is added and a second pressing cycle is applied, the mechanical properties increase as the starch is more completely gelatinised. In general, better properties are achieved with a shorter pressing time and subjecting the boards to a second pressing cycle.

The giant reed rhizome boards obtained consume less energy than those described by other authors [37,47,48,49]. The properties of the rhizome boards manufactured show that it is necessary to continue investigating the different manufacturing parameters to find the most suitable process.

## 4. Conclusions

By improving the manufacturing parameters, it is feasible to manufacture binderless giant reed rhizome particleboards with a reduced energy expenditure (low temperature, pressure and time) while achieving good mechanical properties.

With a longer pressing time, the mechanical properties are improved. This result may be explained by the high concentration of sugars in giant reed rhizomes and their conversion into furfural due to the temperature applied in the press, which significantly favours the self-binding mechanism of the particles.

With a shorter pressing time, better TS and WA properties are achieved. When a second pressing cycle is applied with the addition of water, the mechanical properties are improved without affecting the TS and WA. This may be because the entire starch content of the rhizome is gelatinised. Therefore, in the production of this type of giant reed rhizome board, the properties are improved by keeping them in the hot plate press for a shorter time and subjecting them to two pressing cycles.

Particle size has a very important effect on the mechanical properties of the boards, and it can be concluded that the best mechanical results were achieved with the boards that had particle sizes of 0.25 to 1 mm, which meet the requirements specified in the European standards for type P1 and P2 boards.

The utilisation of these waste materials to manufacture products with a long useful life, such as particleboards, may be beneficial to the environment, as it is a method of carbon fixation; it therefore contributes to reducing CO_2_ in the atmosphere.

## Figures and Tables

**Table 1:** Characteristics of the rhizome particleboards manufactured.

Table	Class	Particle Size (mm)	Temp. (°C)	Time (min)	No. of Boards
B0	B01	0.25 to 1	110	7	3
	B02	0.25 to 1	110	7 + 7	3
B1	B11	0.25 to 1	110	15	3
	B12	0.25 to 1	110	15 + 15	3
C0	C01	1 to 2	110	7	3
	C02	1 to 2	110	7 + 7	3
C1	C11	1 to 2	110	15	3
	C12	1 to 2	110	15 + 15	3
D0	D01	2 to 4	110	7	3
	D02	2 to 4	110	7 + 7	3
D1	D11	2 to 4	110	15	3
	D12	2 to 4	110	15 + 15	3

**Table 2:** Mean values of the physical properties of the binderless giant reed rhizome particleboards.

Type	Class	Density (kg/m^3^)	TS 2 h (%)	TS 24 h (%)	WA 2 h (%)	WA 24 h (%)
B0	B01	812.74 (22.94)	44.69 (7.45)	57.74 (2.70)	69.35 (3.58)	89.00 (3.55)
	B02	882.82 (30.31)	33.36 (12.06)	52.73 (10.23)	66.92 (8.62)	94.85 (0.74)
B1	B11	911.10 (39.31)	33.04 (8.44)	50.52 (4.45)	60.67 (9.30)	81.63 (13.80)
	B12	912.76 (75.03)	37.41 (9.91)	53.98 (6.57)	69.98 (14.73)	87.62 (18.54)
C0	C01	775.54 (44.66)	26.32 (1.78)	43.29 (7.83)	36.28 (9.17)	69.68 (9.82)
	C02	863.44 (98.37)	29.00 (7.96)	44.15 (8.51)	35.54 (9.69)	67.09 (3.61)
C1	C11	735.25 (05.00)	53.43 (0.27)	59.31 (1.55)	85.45 (3.45)	106.12 (5.01)
	C12	799.63 (58.53)	45.84 (6.29)	58.98 (3.64)	93.36 (8.53)	100.96 (6.52)
D0	D01	785.88 (44.90)	37.47 (7.89)	52.65 (11.08)	82.32 (9.28)	104.28 (7.46)
	D02	885.41 (69.17)	28.81 (7.37)	52.19 (10.06)	54.73 (19.79)	88.35 (14.12)
D1	D11	742.80 (72.17)	50.18 (20.98)	60.21 (6.56)	85.06 (10.26)	103.89 (2.28)
	D12	827.48 (38.81)	53.39 (6.95)	72.50 (6.31)	81.00 (6.96)	97.34 (3.33)

TS: thickness swelling. WA: water absorption. (..): standard deviation.

**Table 3:** ANOVA of the results of the tests.

Factor	Properties	Sum of Squares	d.f.	Half Quadratic	F	Sig.
Particle size	Density (kg/m^3^)	48,322.613	2	24,161.306	5.027	0.012
	TS 2 h (%)	118.663	2	59.332	0.376	0.690
	TS 24 h (%)	360.998	2	180.499	20.033	0.147
	WA 2 h (%)	854.916	2	427.548	1.037	0.365
	WA 24 h (%)	973.553	2	486.776	2.430	0.103
	MOR (N/mm^2^)	202.902	2	101.451	26.799	0.000
	MOE (N/mm^2^)	4,264,527.527	2	2,132,010.263	37.620	0.000
	IB (N/mm^2^)	0.154	2	0.077	4.159	0.024
Pressing time	Density (kg/m^3^)	1620.5993	1	1620.5993	0.265	0.610
	TS 2 h (%)	1343.345	1	1343.345	11.380	0.002
	TS 24 h (%)	604.340	1	604.340	7.928	0.008
	WA 2 h (%)	4261.478	1	4261.478	13.885	0.001
	WA 24 h (%)	928.522	1	928.522	4.717	0.037
	MOR (N/mm^2^)	40.153	1	40.153	4.685	0.038
	MOE (N/mm^2^)	599,097.660	1	599,097.660	4.226	0.045
	IB (N/mm^2^)	0.192	1	0.192	12.112	0.001
Pressing cycle	Density (kg/m^3^)	42,416.776	1	42,416.776	8.623	0.012
	TS 2 h (%)	71.939	1	71.939	0.463	0.501
	TS 24 h (%)	51.792	1	51.792	0.560	0.459
	WA 2 h (%)	79.091	1	79.091	0.184	0.671
	WA 24 h (%)	56.475	1	56.475	0.254	0.618
	MOR (N/mm^2^)	39.063	1	39.063	4.706	0.037
	MOE (N/mm^2^)	654,079.258	1	654,079.258	4.180	0.049
	IB (N/mm^2^)	0.080	1	0.080	4.459	0.042

d.f.: degrees of freedom. F: Fisher–Snedecor distribution. Sig.: significance.

**Table 4:** EDS analysis of the chemical composition of different sections of rhizome.

	Section	Chemical Element												
		C	O	Mg	Al	Si	K	Ca	Fe	S	P	Cl	Na	F
**Composition (wt%) of dry matter**	S1	32.40	51.88			15.71								
	S2	31.85	52.26	4.51	1.20	1.74	0.80	6.76	0.38	0.16	0.17	0.16		
	S3	35.30	42.50	6.91			6.27				7.42	0.32	0.80	0.48

**Table 5:** Characteristics of the type of panels manufactured and classification.

	Class	MOR (N/mm^2^)	MOE (N/mm^2^)	IB (N/mm^2^)	TS 24 h (%)
This work	B01	11.56	1667.04	0.58	57.74
	B02	14.24	1903.62	0.75	52.73
	B11	12.02	1603.79	0.95	50.52
	B12	14.00	2052.45	1.12	53.98
	C02	10.52	1378.30	0.72	44.15
[42] (thickness of 6 to 13 mm)	Type P1	10.50	-	0.28	-
	Type P2	11.00	1800.00	0.40	-
	Type P3	15.00	2050.00	0.50	17.00

**Table 6:** Values of the properties obtained by different authors with binderless particleboards.

Reference	Material	Tem. (°C)	Time (min)	Density (kg/m^3^)	TS 24 h (%)	MOR (N/mm^2^)	MOE (N/mm^2^)	IB (N/mm^2^)
[46]	Date palm	180	2	1200	150.00	8.40	928.00	0.13
[47]	Oil palm	180	20	800	20.00	13.57		0.71
[48]	Canary Islands palm	120	30	850	27.56	13.00	1467.82	0.40
[37]	Rice straw	110	30 + 30	1140	53.75	15.09	2696.85	0.18
This study	Giant reed rhizome	110	7 + 7	883	52.73	14.24	1903.62	0.75
